# Assessment of Selected Matrix Metalloproteinases (MMPs) and Correlation with Cytokines in Psoriatic Patients

**DOI:** 10.1155/2021/9913798

**Published:** 2021-06-29

**Authors:** Anna Michalak-Stoma, Joanna Bartosińska, Dorota Raczkiewicz, Małgorzata Kowal, Dorota Krasowska, Grażyna Chodorowska

**Affiliations:** ^1^Chair and Department of Dermatology, Venereology and Pediatric Dermatology, Medical University of Lublin, ul. Staszica 16, 20-081 Lublin, Poland; ^2^Department of Cosmetology and Aesthetic Medicine, Medical University of Lublin, ul. Chodźki 1, 20-093 Lublin, Poland; ^3^Department of Medical Statistics, School of Public Health, Center of Postgraduate Medical Education, ul. Kleczewska 61/63, 01-826 Warsaw, Poland

## Abstract

Metalloproteinases (MMPs) and cytokines have a great impact on the pathogenesis of psoriasis. Cytokines, as key mediators of inflammation and autoimmune processes, play a crucial role in the regulation of MMP expression in different cell types. Parallel, MMPs have an influence on cytokine production. This interaction was not well recognized in psoriatic patients. Our study is aimed at assessing the selected serum MMP levels and their correlations with cytokine levels in the serum of psoriatic patients. We observed a significantly elevated level of pro-MMP-1 and MMP-9 in psoriatic patients' serum in comparison to the control group. We did not observe any statistically significant differences of MMP-3 and pro-MMP-10 between the psoriatic patients and the control group. We did not observe any statistically significant differences in all the studied MMP levels between the patients with and without psoriatic arthritis (PsA). MMP-3 level correlated positively with proinflammatory cytokines, i.e., IL-12p/70, IL-17A, and TNF-*α* as well as MMP-3 and pro MMP-1 correlated positively with IL-4 in the psoriatic patients. In the control group, a positive correlation between pro-MMP-1 and TNF-*α* was found. These results confirm MMPs and Th1 and Th17 cytokine interaction in the inflammatory regulation in psoriasis.

## 1. Introduction

Psoriasis is a frequent skin disease whose pathogenesis is still not fully understood. Matrix metalloproteinases (MMPs) are a family of proteolytic enzymes involved in many physiological processes like tissue remodeling, cell migration, angiogenesis, and epithelial apoptosis [1]. According to MMP structure, substrate specificity, and function, MMPs can be divided into 9 subgroups: collagenases (MMP-1, MMP-8, and MMP-13), gelatinases (MMP-2 and MMP-9), stromelysins (MMP-3 and MMP-10), stromelysin-like MMPs (MMP-11 and MMP-12), matrilysins (MMP-7 and MMP-26), transmembrane MMPs (MMP-14, MMP-15, MMP-16, and MMP-24), glycosylphosphatidylinositol- (GPI-) type MMPs (MMP-17 and MMP-25), MMP-19-like MMPs (MMP-19 and MMP-28), and other MMPs (MMP-18, MMP-20, and MMP-23) [2, 3]. Many metalloproteinases are involved in psoriasis pathogenesis. MMP-1, MMP-2, MMP-3, MMP-8, MMP-9, MMP-12, and MMP-19 influence the migration of epidermal keratinocytes in the psoriatic epidermis [1, 4]. MMP-1 induction increases also the mobility of dermal fibroblasts [1, 5]. High MMP-9 levels can inhibit the mobility of dermal fibroblasts and epidermal keratinocytes [6, 7] and can slow down skin healing [8]. The correct balance between the different MMPs is also important for microcapillary permeability. The changes in MMP-2, MMP-3, MMP-9, and MMP-12 expression in psoriatic skin may indicate its predisposition to the growth of new capillaries [1, 9, 10]. Many MMPs are involved in the regulation of inflammatory response in psoriasis. The infiltration of lymphocytes follows the presence of monocytes, macrophages, and neutrophils in the epidermis. Monocytes express MMP-1, MMP-7, MMP-8, and MMP-9; macrophages express MMP-1, MMP-2, MMP-3, MMP-8, MMP-9, MMP-10, MMP-12, and MMP-13; and neutrophils secrete MMP-8, MMP-9, and MMP-25 [1, 11]. The activation of distinct T cell subsets drives the maintenance phase of psoriatic inflammation. Especially, the Th1 cytokines, interferon- (IFN-) *γ*, tumor necrosis factor- (TNF-) *α*, and interleukin- (IL-) 12, and Th17 cytokines, IL-17, IL-21, IL-22, IL-25, IL-26, and TNF-*α*, are responsible for keratinocyte proliferation and the maintenance of inflammation. Keratinocytes participate actively in the inflammatory cascade through cytokine (IL-1, IL-6, IFN-*γ*, and TNF-*α*), chemokine, and antimicrobial peptide (AMP) secretion [12]. All these processes lead to tissue remodeling in psoriasis.

Our study is aimed at assessing the selected serum MMP levels and their correlations with cytokine levels in the serum of psoriatic patients.

## 2. Materials and Methods

### 2.1. Studied Group

The study was conducted in patients hospitalized in the Department of Dermatology, Venereology and Pediatric Dermatology, Medical University of Lublin, Poland, because of psoriasis exacerbation. The study comprised 58 male psoriatic patients and 29 male healthy controls. 36% of patients with coexisting joint problems met the Classification of Psoriatic Arthritis (CASPAR) criteria for psoriatic arthritis (PsA). All PsA patients had a polyarticular, asymmetrical subset of the disease (more than 5 joints were affected). Demographic data, medical history, and serum for assessment of the selected MMPs and cytokines were collected from all participants.

### 2.2. Assessment of Psoriasis Severity

The skin lesion severity was assessed with the use of PASI (Psoriasis Area and Severity Index), BSA (Body Surface Area), and PGA (Physician Global Assessment) scores.

### 2.3. Assessment of MMPs' Serum Concentrations in Psoriatic Patients and Controls

Blood samples were collected from psoriatic patients and controls and were centrifuged for 15 minutes at 1000 x g. Then, serum samples were subdivided into small aliquots to be stored at -80°C until tested for MMP levels. In the studied psoriatic patients as well as the control group, the concentrations of pro-MMP-1, MMP-3, MMP-9, and pro-MMP-10 were determined with the use of R&D Systems kits (R&D Systems, Minneapolis, MN, USA), according to the manufacturer's instructions.

### 2.4. Assessment of Cytokines' Serum Concentrations in Psoriatic Patients and Controls

The concentrations of IL-12p70, IFN-*γ*, IL-17A, IL-2, IL-10, IL-9, IL-22, IL-6, IL-13, IL-4, IL-5, IL-1beta, and TNF-*α* were detected with eBioscience Th Cell Differentiation Th1/Th2/Th9/Th17/Th22 FlowCytomix™ Multiple Analyte Detection System according to the manufacturer's protocol.

The study was approved by the Polish Local Ethics Committee.

### 2.5. Statistical Analyses

Statistical analysis was performed using the STATISTICA software. Mean values (*M*) and standard deviation (SD) were calculated for continuous variables or absolute number (*n*) and relative number (%) of occurrences of items for categorical variables. The following statistical tests were applied: Student's *t*-test to compare age, MMPs, and cytokine levels between study and control groups; Mann-Whitney's *U* test to compare MMPs and cytokines in patients with and without PsA; and Pearson's *r* correlation coefficient to correlate MMPs with cytokines as well as to correlate MMPs and cytokines with severity of psoriasis. In all statistical tests, the level of significance was set at 0.05.

## 3. Results and Discussion

### 3.1. Sociodemographic Characteristics

Sociodemographic characteristics of psoriasis patients are presented in Table 1.

The control group's age (min–max 26-72, *M* ± SD47.3 ± 11.8) did not significantly differ from the studied psoriatic patients' age (*p* = 0.842).

### 3.2. Serum MMP and Cytokine Concentrations in Psoriatic Patients and Control Group

Comparison of metalloproteinases and cytokines between psoriatic patients and control group is presented in Table 2. We observed a significantly elevated level of pro-MMP-1 and MMP-9 in psoriatic patients' serum in comparison to the control group (*p* = 0.006 and *p* = 0.017, respectively). In a healthy skin, MMP-9 is expressed in keratinocytes and immune cells, like lymphocytes, macrophages, eosinophils, and mast cells [13]. Neutrophils synthesize MMP-9 during their maturation in the bone marrow and store it in specific neutrophil granules. MMP-9 secretion is stimulated by a variety of external stimuli and is associated with the activation of cells. Loss of MMP-9 prolongs inflammation in contact hypersensitivity [2, 14]. In previous studies, MMP-1 and MMP-9 expression was observed to be significantly elevated in a psoriatic skin [15–18]. High MMP-1 and MMP-9 levels were also noticed in psoriatic patients' serum [19–23]. Choi et al. [24] demonstrated that NB-UVB irradiation upregulated MMP-1 expression at both the mRNA and protein levels. However, MMP-3 and MMP-9 were decreased after NB-UVB treatment [22]. Anti-TNF treatment decreased blood levels of MMP-1 and MMP-9 [20, 25]. MMP-9 lesional and serum levels were also reduced after therapy with anti-TNF in the study of Cordiali-Fei et al. [26].

We did not observe any statistically significant differences in MMP-3 and MMP-10 between the psoriatic patients and the control group (*p* = 0.391 and *p* = 0.256, respectively). In previous studies, MMP-3 was not detected in a healthy skin [27]. In a psoriatic skin, MMP-3 level was positively correlated with the level of the proinflammatory cytokine IL-22 [28]. MMP-3 appears to be essential for skin inflammation [2]. Elevated level of MMP-3 was discovered in the serum from patients with psoriasis compared to the healthy controls [22, 29]. MMP-10 was not detected in the psoriatic skin [16]. Diani et al. [23] observed elevated serum MMP-10 levels in psoriatic patients. It was noticed that the serum concentration of MMP-3 in PsA patients decreased after anti-TNF-*α* treatment [25, 30–33].

The mean fluorescence intensity of IFN-*γ*, IL-10, IL-9, and IL-13 was not intensive enough to calculate the cytokine concentration. In the psoriatic patients, IL-6 level was higher than in the control group and this difference was statistically significant (*p* = 0.05). The results were in accordance with previous studies which demonstrated the increased IL-6 serum level in psoriatic patients compared to healthy controls [34–37]. The decrease of serum IL-6 level was observed after MTX treatment [36], UVB radiation, topical steroids, infliximab, and adalimumab [38, 39]. On the other hand, ustekinumab did not affect serum IL-6 levels [39]. We did not observe any statistically significant differences in other studied cytokine levels between the psoriatic patients and control group.

### 3.3. Serum MMP and Cytokine Concentrations in Psoriatic Patients with and without Arthritis

Comparison of metalloproteinases and cytokines between psoriatic patients with and without arthritis is presented in Table 3. We did not observe any statistically significant differences in the analyzed MMP and cytokine levels between the psoriatic patients with and without arthritis. However, some authors recognized MMP-1 and MMP-3 as the biomarkers of PsA [25, 29, 40–42]. Diani et al. [23] observed elevation of serum MMP-1, MMP-9, and MMP-10 both in psoriatic patients and in patients with PsA.

### 3.4. Correlations of MMPs and Cytokines with Psoriasis Severity

Correlations of MMP and cytokine levels with severity of psoriasis are presented in Table 4. In our study, only MMP-3 levels correlated negatively with the severity of psoriasis measured by PASI and sPGA. There are not many studies to compare these results. Diani et al. [23] observed no statistically significant correlations between MMP-1, MMP-3, and MMP-9 levels and duration of psoriasis or PsA as well as the severity of the disease calculated with PASI. Flisiak et al. [19] reported that the elevated levels of MMP-1 in psoriatic plasma and the levels of MMP-1 were inversely correlated to disease severity. The correlation between the levels of MMP-9 and the severity of disease was noticed by Buommino et al. [20]. Serum concentration of MMP-10 presented a negative correlation with PsA duration and a positive correlation with PASI in psoriatic patients [23].

### 3.5. Correlations between MMPs and Cytokines

**Figure 1:**
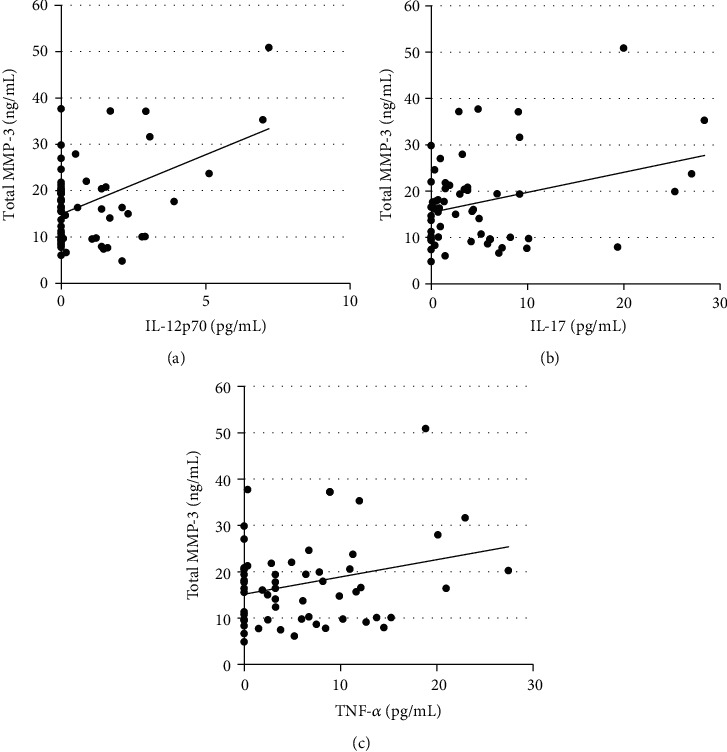
Correlations of total MMP-3 with (a) IL-12p70, (b) IL-17A, and (c) TNF-*α*.

Correlations between MMPs and cytokines in psoriatic patients and in the control group are presented in Table 5. Positive correlations between MMP-3 level and proinflammatory cytokines IL-12p/70, IL-17A, and TNF-*α* were observed in the psoriatic patients (*p* = 0.001, *p* = 0.017, and *p* = 0.043, respectively, Figure 1). MMP-3 and pro-MMP-1 correlated positively with IL-4 in the psoriatic patients (*p* = 0.001 and *p* = 0.037, respectively). In the control group, a positive correlation between pro-MMP-1 and TNF-*α* was found (*p* < 0.001).

It is already recognized that both MMPs and cytokines have the great impact on the pathogenesis of psoriasis [1, 2, 12]. Cytokines, as key mediators of inflammation and autoimmune processes, play a crucial role in the regulation of MMP expression in different cell types [13, 43, 44]. It was noticed that MMP-1 is upregulated by IL-1beta and TNF-*α*. IL-4 has an inhibitory effect, whereas IFN-*γ* and IL-6 can present both stimulating and downregulating actions [44]. MMP-3 and MMP-10 production is elevated by IL-1beta and TNF-*α*. TGF-beta and IFN-*γ* presented the inhibitory effect. The upregulation of MMP-9 was observed by TGF-beta, TNF-*α*, IFN-*γ*, and IL-1beta [18, 43, 45, 46]. Some studies have also shown induction of MMP-9 protein expression by IL-18 [47, 48]; others did not observe the IL-18 influence on MMP-9 expression [43]. Anti-inflammatory Th2 cytokines, such as IL-4 and IL-10, are reported to downregulate MMP-9 [43, 47, 49]. IL-12 was shown to downregulate MMP-9 in stromal cells [50] and has no effect on MMP-9 induction in peripheral blood monocytes or T cells [43, 47]. Elevated IL-18, IL-12, and TNF-*α* levels observed in autoimmune disorders may influence the increased MMP activity associated with these conditions. On the other hand, MMPs have also an impact on the inflammatory process by activation of TGF-beta or degradation of IL-1beta, as well as by releasing TNF-*α* or by influence on cytokine receptors such as IL-6 and TGF-alpha [43].

We decided to study the correlations between Th1/Th2/Th9/Th17/Th22 cytokines and selected MMPs, because the understanding of the cytokine and MMP interactions is essential for better diagnosis and treatment of psoriasis, which is nowadays recognized as autoimmune and autoinflammatory disease. The results of Amezcua-Guerra et al. [51] suggest that sera from patients with psoriasis may influence the response of monocytes to stimulation with IFN-*γ* observed as the increased production of MMP-9. These results suspect the existence of a primed state in inflammatory cells of psoriatic patients [51]. However, according to the PubMed search, there are no studies comparing the levels of MMPs and cytokines in psoriatic patients. There are only a few studies comparing the levels of MMP-1, MMP-3, or MMP-9 before and after anti-TNF treatment [20, 25, 26, 30–33]. The decrease of MMPs after anti-TNF treatment can suggest the important influence of TNF-*α* on MMPs' production.

## 4. Conclusions

Metalloproteinases and cytokines have the great impact on the pathogenesis of psoriasis. However, interactions between them in psoriasis are not as well recognized as in other autoimmune disorders. Positive correlations between MMP-3 level and proinflammatory cytokines IL-12p/70, IL-17A, and TNF-*α* recognized in our study confirm MMPs and Th1 and Th17 cytokines' cross talk in the inflammatory regulation in psoriasis.

## Figures and Tables

**Table 1:** Clinical data of psoriatic patients.

	Study group (*N* = 58)
Age (years), min–max, *M* ± SD	26-73, 46.7 ± 13.9
Duration of psoriasis (years), min–max, *M* ± SD	1-45, 20.9 ± 12.0
Age of psoriasis onset (years), min–max, *M* ± SD	8-55, 25.9 ± 10.3
Duration of psoriatic arthritis, min–max, *M* ± SD	1-25, 10.0 ± 6.3
Positive family history, *n* (%)	21 (36.21)
PASI, min–max, *M* ± SD	8-57, 23.6 ± 12.0
BSA (%), min–max, *M* ± SD	4-85, 27.9 ± 20.8
PGA, *n* (%)	
2	8 (13.79)
3	33 (56.90)
4	12 (20.69)
5	5 (8.62)

**Table 2:** Comparison of metalloproteinases and cytokines between psoriatic patients and control group. ∗ indicates statistically significant differences.

Metalloproteinase or cytokine	Study group (*N* = 58)	Control group (*N* = 29)	*p*
Pro-MMP-1 (ng/mL)	7.75 ± 4.48	5.08 ± 3.47	0.006^∗^
Total MMP-3 (ng/mL)	17.52 ± 9.39	19.22 ± 7.08	0.391
MMP-9 (ng/mL)	1165.16 ± 472.93	903.08 ± 473.27	0.017^∗^
Pro-MMP-10 (pg/mL)	492.98 ± 229.46	554.76 ± 253.69	0.256
IL-12p70 (pg/mL)	1.01 ± 1.65	0.92 ± 1.43	0.822
IL-17A (pg/mL)	4.83 ± 6.75	3.2 ± 5.11	0.256
IL-2 (pg/mL)	18.87 ± 20.41	13.72 ± 18.29	0.255
IL-22 (pg/mL)	169.28 ± 65.74	163.46 ± 98.60	0.744
IL-6 (pg/mL)	1.41 ± 1.55	0.79 ± 0.99	0.050^∗^
IL-4 (pg/mL)	1.26 ± 2.69	0.93 ± 1.95	0.560
IL-5 (pg/mL)	1.72 ± 2.69	1.49 ± 2.62	0.700
IL-1beta (pg/mL)	14.35 ± 26.95	25.41 ± 39.85	0.130
TNF-*α* (pg/mL)	6.35 ± 6.72	7.36 ± 7.95	0.535

**Table 3:** Comparison of metalloproteinases and cytokines between psoriatic patients with and without arthritis.

Metalloproteinase or cytokine	Psoriasis arthritis (*N* = 21)	Psoriasis vulgaris (*N* = 37)	*p*
Pro-MMP-1 (ng/mL)	7.75 ± 3.61	7.75 ± 4.96	0.633
Total MMP-3 (ng/mL)	18.58 ± 11.34	16.91 ± 8.19	0.821
MMP-9 (ng/mL)	1243.84 ± 610.65	1120.51 ± 375.92	0.728
Pro-MMP-10 (pg/mL)	545.63 ± 290.93	463.10 ± 183.86	0.523
IL-12p70 (pg/mL)	1.25 ± 2.10	0.87 ± 1.34	0.619
IL-17A (pg/mL)	5.81 ± 7.07	4.28 ± 6.60	0.236
IL-2 (pg/mL)	23.31 ± 24.35	16.34 ± 17.67	0.226
IL-22 (pg/mL)	175.47 ± 64.79	165.76 ± 66.91	0.610
IL-6 (pg/mL)	1.21 ± 1.27	1.52 ± 1.70	0.484
IL-4 (pg/mL)	1.39 ± 2.99	1.18 ± 2.54	0.786
IL-5 (pg/mL)	1.57 ± 1.78	1.80 ± 3.11	0.336
IL-1beta (pg/mL)	12.33 ± 25.12	15.50 ± 28.21	0.654
TNF-*α* (pg/mL)	6.46 ± 5.74	6.29 ± 7.29	0.540

**Table 4:** Correlations between metalloproteinases and cytokines and severity of psoriasis (*N* = 58). ∗ indicates statistically significant differences.

Metalloproteinase or cytokine		PASI	BSA	sPGA
Pro-MMP-1 (ng/mL)	*r*	0.087	0.065	0.157
	*p*	0.517	0.631	0.241

Total MMP-3 (ng/mL)	*r*	-0.286	-0.196	-0.264
	*p*	0.030^∗^	0.141	0.045^∗^

MMP-9 (ng/mL)	*r*	-0.034	0.017	0.097
	*p*	0.800	0.898	0.468

Pro-MMP-10 (pg/mL)	*r*	-0.209	-0.147	-0.013
	*p*	0.116	0.270	0.920

IL-12p70 (pg/mL)	*r*	-0.154	-0.034	-0.092
	*p*	0.249	0.799	0.492

IL-17A (pg/mL)	*r*	-0.069	-0.098	-0.058
	*p*	0.608	0.464	0.667

IL-2 (pg/mL)	*r*	-0.076	-0.102	-0.094
	*p*	0.571	0.445	0.484

IL-22 (pg/mL)	*r*	-0.035	-0.113	-0.175
	*p*	0.794	0.398	0.189

IL-6 (pg/mL)	*r*	-0.090	-0.021	-0.101
	*p*	0.500	0.879	0.452

IL-4 (pg/mL)	*r*	-0.140	-0.083	-0.154
	*p*	0.295	0.537	0.248

IL-5 (pg/mL)	*r*	0.240	0.248	0.240
	*p*	0.069	0.060	0.070

IL-1beta (pg/mL)	*r*	0.115	0.092	0.167
	*p*	0.390	0.493	0.212

TNF-*α* (pg/mL)	*r*	-0.157	-0.120	-0.157
	*p*	0.239	0.370	0.238

**Table 5:** Correlations between metalloproteinases and cytokines in psoriatic patients and in control group. ∗ indicates statistically significant differences.

Cytokine		Study group (*N* = 58)				Control group (*N* = 29)			
		Pro-MMP-1 (ng/mL)	Total MMP-3 (ng/mL)	MMP-9 (ng/mL)	Pro-MMP-10 (pg/mL)	Pro-MMP-1 (ng/mL)	Total MMP-3 (ng/mL)	MMP-9 (ng/mL)	Pro-MMP-10 (pg/mL)
IL-12p70 (pg/mL)	*r*	0.091	0.450	0.038	0.114	-0.177	-0.177	-0.251	-0.074
	*p*	0.500	0.001^∗^	0.776	0.393	0.357	0.358	0.189	0.702

IL-17A (pg/mL)	*r*	0.229	0.312	0.029	-0.034	-0.070	0.126	0.031	0.171
	*p*	0.084	0.017^∗^	0.828	0.800	0.717	0.514	0.873	0.375

IL-2 (pg/mL)	*r*	-0.108	0.119	-0.194	0.190	0.133	0.092	-0.127	-0.023
	*p*	0.419	0.374	0.144	0.152	0.491	0.635	0.511	0.907

IL-22 (pg/mL)	*r*	-0.032	0.177	-0.174	-0.124	0.069	-0.029	0.143	0.172
	*p*	0.812	0.185	0.193	0.356	0.722	0.883	0.460	0.372

IL-6 (pg/mL)	*r*	0.153	0.152	-0.003	0.136	0.186	-0.042	0.211	-0.066
	*p*	0.253	0.254	0.980	0.309	0.334	0.828	0.271	0.733

IL-4 (pg/mL)	*r*	0.274	0.414	0.068	-0.011	-0.067	0.016	-0.098	0.228
	*p*	0.037^∗^	0.001^∗^	0.614	0.936	0.728	0.933	0.614	0.234

IL-5 (pg/mL)	*r*	0.133	-0.101	0.008	-0.166	-0.066	0.277	-0.014	-0.128
	*p*	0.320	0.450	0.951	0.212	0.734	0.146	0.941	0.507

IL-1beta (pg/mL)	*r*	0.012	0.113	0.068	0.054	0.205	0.169	-0.031	-0.185
	*p*	0.931	0.401	0.611	0.688	0.286	0.381	0.875	0.337

TNF-*α* (pg/mL)	*r*	0.071	0.267	-0.059	0.045	0.645	0.023	-0.122	-0.169
	*p*	0.599	0.043^∗^	0.663	0.738	<0.001^∗^	0.905	0.530	0.382

## Data Availability

The data used in this study are available from the corresponding author upon request.
